# A Novel Relative Permeability Model for Gas and Water Flow in Hydrate-Bearing Sediments With Laboratory and Field-Scale Application

**DOI:** 10.1038/s41598-020-62284-5

**Published:** 2020-03-30

**Authors:** Harpreet Singh, Evgeniy M. Myshakin, Yongkoo Seol

**Affiliations:** 1 National Energy Technology Laboratory, Morgantown, WV USA; 2 LRST, 626 Cochrans Mill Road, Pittsburgh, PA USA

**Keywords:** Hydrology, Natural gas

## Abstract

In a producing gas hydrate reservoir the effective porosity available for fluid flow constantly changes with dissociation of gas hydrate. Therefore, accurate prediction of relative permeability using legacy models (*e.g*. Brooks-Corey (B-C), van Genuchten, etc.) that were developed for conventional oil and gas reservoirs would require empirical parameters to be calibrated at various *S*_*h*_ over its range of variation, but such calibrations are precluded because of lack of experimental relative permeability data. This study proposes a new relative permeability model for gas hydrate-bearing media that is a function of maximum capillary pressure, capillary entry pressure, pore size distribution index, residual saturations, hydrate saturation, and four other constants. The three novel features of the proposed model are: (i) requires fitting its six empirical parameters only once using experimental data from any single *S*_*h*_, and the same set of empirical parameters predict relative permeability at all *S*_*h*_, (ii) includes the effect of capillarity, and (iii) includes the effect of pore-size distribution. From practical standpoint, the model can be used to simulate multiphase flow in gas hydrate-bearing sediments where the proposed relative permeability can account for the evolving hydrate saturation. The proposed model is implemented in a numerical simulator and the wall time required to perform simulations using the proposed model is shown to be similar to the time it takes to run same simulations with the B-C model. The proposed model is a step forward towards achieving the goal of physically accurate modeling of multiphase flow for gas hydrate-bearing sediments that accounts for the effect of gas hydrate saturation change on relative permeability.

## Introduction

Methane hydrate is a vast resource of methane gas^1–5^ that holds a potential to supply natural gas at industrial-scale to meet the ever increasing energy demand of the world. Currently, for a producing gas hydrate reservoir, understanding the dynamic nature of the relative permeability is challenging and difficult due to lack of experimental datasets in the literature for simultaneous two-phase fluid flow in gas hydrate-bearing sediments. The experimental datasets on permeability existing in the literature^6–10^ are varying permeability of single phase flow (water) as a function of hydrate saturation, which is also referred by many researchers as ‘permeability reduction curve’. Although relative permeability of conventional reservoir rocks has been studied for a long time^11,12^, to our knowledge, there seems to be no peer-reviewed literature on experimental relative permeability of combined gas and brine flow in hydrate-bearing sediments. Numerical simulations of gas hydrate uses empirical models^13–16^ (*e.g*. B-C, van Genuchten, etc.) that were originally developed for conventional oil and gas reservoirs. The empirical parameters of these models assume that porous media properties (e.g. rock heterogeneity, rock properties, etc.) do not change during production. However, in a producing gas hydrate reservoir, the effective porosity available for fluid flow constantly changes with dissociation of gas hydrate^17,18^, meaning that accurate prediction of relative permeability using such models would require empirical parameters to be a function of *S*_*h*_. Practically, this may involve calibrating empirical parameters at various *S*_*h*_ over its range of variation, but because of lack of experimental relative permeability data such calibrations are precluded.

To overcome the lack of experimental relative permeability data for gas hydrate, we recently proposed a mechanistic model^19,20^ for hydrate-bearing media that is based on physics and predicts relative permeability without the need for calibrations at various *S*_*h*_. Although this mechanistic model^19,20^ can overcome the need for calibrations at various *S*_*h*_, it may be computationally more expensive to use in a reservoir simulator than existing empirical models. The model proposed in this study, while overcoming the need for calibrations at various *S*_*h*_, is expected to be computationally less expensive than mechanistic model^19,20^ and additionally it can account for heterogeneity in pore-sizes that is not possible using the mechanistic model.

This study proposes a novel relative permeability model developed specifically for hydrate-bearing porous media with multiphase flow of gas and water. Therefore, the model is referred, in short, as RPHCP for relative permeability of hydrate-bearing media with capillarity and pore-size distribution. Although relative permeability models of conventional reservoir rocks have been proposed with the effect of capillarity and pore-size distribution^11,12,21,22^, these models were not meant for gas hydrate as they do not account for evolution of permeability and multiphase flow as a result of dissociating gas hydrate. The approach used in deriving RPHCP, which is an analytical function of maximum capillary pressure, capillary entry pressure, pore size distribution index, hydrate saturation, and residual saturations, is discussed next. Next, RPHCP is validated against gas and water relative permeability data reported by Mahabadi *et al*.^23^ who used pore-scale simulations in gas hydrate-bearing sediments recovered from the gas hydrate deposit at the Mallik Site. The model is then used in a numerical simulator and compared for its computational expense against the B-C model, followed by a discussion on the practical implications of using the proposed model in simulations of gas hydrate-bearing media.

## Model

Although correct prediction of relative permeability is central to multiphase flow studies in subsurface and other industrial applications, there is no standard method by which relative permeability is estimated. Typically, for lack of theoretical understanding and physics-based models, relative permeability is estimated through laboratory experiments, and the experimental data is then often fitted to one of the many empirical models to predict relative permeability for that rock. One such empirical model to predict relative permeability was proposed by Purcell^24^ who developed an equation to estimate rock permeability by using capillary pressure data. The equation proposed by Purcell^24^ can be readily extended to different multiphase flow problems, and that equation is used as a basis to develop the model for gas hydrate-bearing sediments in this study.

### Approach

The approach to derive relative permeability of hydrate-bearing sediments is based on the equation by Purcell^24^ who proposed an approach to infer rock permeability using capillary pressure data, which can be directly extended to estimate multiphase relative permeability for a water wet rock as follows:

1 $${k}_{rw}({S}_{w})=\frac{{\int }_{0}^{{S}_{w}}\frac{d{S}_{w}}{{P}_{c}^{2}}}{{\int }_{0}^{1}\frac{d{S}_{w}}{{P}_{c}^{2}}},\,{k}_{rg}({S}_{w})=\frac{{\int }_{{S}_{w}}^{1}\frac{d{S}_{w}}{{P}_{c}^{2}}}{{\int }_{0}^{1}\frac{d{S}_{w}}{{P}_{c}^{2}}}$$

where, *k*_*rw*_, *k*_*rg*_, *S*_*w*_, *P*_*c*_ are relative permeability of water, relative permeability of gas, saturation of water, and capillary pressure, respectively. This equation is used to estimate relative permeability of water with two-phase flow in conventional reservoir rocks, but this equation cannot be directly used for other flow problems where more than two phases are present or where water is not the wetting phase. Therefore, the model proposed in this study is motivated by this simple equation that is modified appropriately to account for the boundary conditions of multiphase flow in gas hydrate-bearing media.

### Capillary pressure model

Capillary pressure data is generally not available for most cases, and in such cases the capillary pressure term is often assumed to be a generic empirical function of saturation. However, even for cases where capillary pressure data is available, the standard practice is to fit data to a pre-selected empirical model for capillary pressure. Similarly, for the purpose of developing an analytical relative permeability model for gas hydrate per the Purcell’s approach, a capillary pressure equation that can be integrated is required. Many studies have proposed different methods to predict capillary pressure, including analytical^25–28^ and numerical methods^29^, but the choice of a capillary function used in this study is based on two requirements, which are: (i) it can be integrated analytically, and (ii) it is applicable to a porous medium. Based on these two conditions, a general capillary pressure model reported by Li^25^ is used as an expression to derive the proposed relative permeability model for gas hydrate-bearing sediments. Even though there are more popular capillary pressure models (e.g. van Genuchten, B-C, etc.), they are either meant for unconsolidated porous media (e.g. van Genuchten) or for consolidated media (e.g. B-C), but the model proposed by Li^25^ is a generalized model that can work in both the consolidate and unconsolidated rocks. The capillary pressure term, shown below, was derived by Li^25^ using a fractal modeling technique and is applicable to porous media:

2 $${P}_{c}={p}_{max}{(1-b{S}_{wn})}^{-\frac{1}{\lambda }},$$

where,

3 $$b=1-{\left(\frac{{P}_{e}}{{P}_{max}}\right)}^{-\lambda }$$

4 $${S}_{wn}=\frac{{S}_{w}-{S}_{wr}}{1-{S}_{gr}-{S}_{wr}}$$

Here, ${P}_{c},{p}_{max},\,{p}_{e},\lambda ,\,{S}_{wn},\,{S}_{w},\,{S}_{wr},\,{S}_{gr}$ represent capillary pressure, capillary pressure at the residual saturation (residual nonwetting-phase saturation in the imbibition case and the residual wetting-phase saturation in the drainage case), capillary entry pressure for the rock (the minimum pressure required to force gas into a water-wet rock during drainage), pore size distribution index, normalized water saturation, water saturation, residual water saturation, and residual gas saturation, respectively. The parameter *b* is a factor that is a function of capillary entry pressure and maximum capillary pressure as shown by Eq. (3). Here, pore size distribution index *(λ*) characterizes the heterogeneity of rocks and using fractal theory it can be related to fractal dimension (*D*_*f*_) as $\lambda =3-{D}_{f}$, where *D*_*f*_ is often less than 3^30^, but it can be higher than 3 for highly heterogeneous media^26,31–34^ such as coal, Geysers rock, etc. In terms of *λ*, this means that porous media with larger heterogeneity have smaller values of *λ* and vice versa^30,35^. The capillary pressure shown above and used in deriving the proposed relative permeability model is a function of gas hydrate saturation, *S*_*h*_, as ${S}_{w}=1-({S}_{h}+{S}_{g})$, therefore, the capillary pressure equation can be written as follows:

5 $${P}_{c}={p}_{max}{\left[1-b\left\{\frac{1-({S}_{h}+{S}_{g})-{S}_{wr}}{1-{S}_{gr}-{S}_{wr}}\right\}\right]}^{-\frac{1}{\lambda }}$$

Dissociation of gas hydrate leads to changes in curvature of the pores that also control capillary pressure to some extent, therefore, capillary pressure changes with *S*_*h*_.

Ideally, for accurate estimation, the capillary pressure of fluids in gas hydrate-bearing media must be delineated into capillary pressure of fluids in contact with gas hydrate and capillary pressure of fluids in contact with rock matrix; however, it may not be possible to develop such a model with the existing experimental data^36^ that provides an averaged capillary pressure without delineation of fluid contact.

#### Brooks-corey and other capillary pressure models

In the case when ${p}_{max}\to \infty$ and *λ* > 0 (similar to *D*_*f*_ < 3), $b\to 0$, the above capillary pressure equation reduces to a limiting case of the widely accepted B-C model^37^. Since the B-C model has been widely used to fit capillary pressure data for many real rock samples, one can see that the above capillary pressure equation can be used to fit even those rocks where the B-C model may not fit well. Similarly, the capillary pressure model discussed above, which would be used to develop a new relative permeability model for gas hydrate, can be reduced to other forms^25^, such as capillary pressure for imbibition and drainage, capillary pressure for a single capillary tube, etc.

### Derivation of relative permeability model

Gas hydrate morphologies are widely described in terms of pore filling (PF), grain coating, load bearing, etc.^3,38–41^, especially for pore-scale studies. Field-scale studies indicate that in sandy gas hydrate deposits a dominant precipitation habit of gas hydrate is PF^3,39^. Most natural core samples show hydrate morphology as PF, plus the validation of models using field-scale data (e.g. Behseresht and Bryant^42^) have also provided evidence that gas hydrate is PF-dominated. Other precipitation habits also exist especially at high gas hydrate saturations, but for practical purposes and for field-scale studies assuming PF gas hydrate is a reasonable approximation. An equivalent relative permeability model for other morphologies can be also derived as shown for grain coating gas hydrate in Appendix (supplementary information). The hydrate morphology is independent of the pore shape and gas hydrate can form in highly tortuous pore space. The derived model considers cylindrical pore shape for simplification. The conceptual model and the boundary conditions required to derive the proposed model are discussed next.

#### Derivation

**Figure 1:**
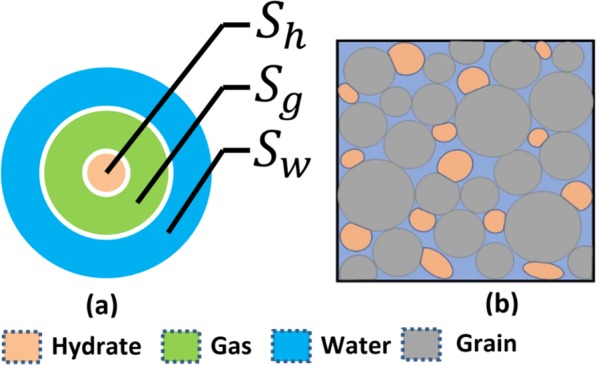
A sketch depicting (**a**) a simplified capillary-shaped cross-section for pore filling hydrate with fluids, and (**b**) its continuum-scale representation in porous media with matrix (grains). The boundary conditions shown by this illustration are independent of the pore shape.

PF gas hydrate is schematically shown in Fig. 1. Under continuous production, this type of hydrate morphology leaves an open annulus such that the wetting phase (water) is close to pore walls^43–45^. Based on experimental observations^43–45^ it has been found that gas hydrate first forms at the grain boundaries and fills pore space as shown in Fig. 1(b). The conceptual model, depicting a cross-section of cylindrical pore space with PF gas hydrate and the products of gas hydrate dissociation, is illustrated in Fig. 1(a), whereas its continuum-scale representation in porous media with matrix (grains) before production is shown in Fig. 1(b). Experimentally, pore-filling hydrate has been shown to naturally become load-bearing hydrate when the pore saturation exceeds approximately 40%^3^. Hydrate formation models have been developed based on different hypothesis^42,46–54^ describing how hydrates are formed, which suggests lack of conclusive evidence on the mechanism behind formation of hydrates. In this study the conceptual model presented in Fig. 1(a) implies that gas hydrate is already formed in porous media and continuous gas production leads to hydrate decomposition. Further discussion related to the distribution of fluids around gas hydrates and its effect on relative permeability can be found in our recent works^19,20^.

##### Model

Gas and water relative permeability are derived by using the boundary conditions from Fig. 1(a) within the limits of the integral as follows:

6 $${k}_{rg}=\frac{{\int }_{0}^{{S}_{h}+{S}_{g}-{S}_{gr}}\frac{d{S}_{w}}{{P}_{c}^{2}}-{\int }_{0}^{{S}_{h}}\frac{d{S}_{w}}{{P}_{c}^{2}}}{{\int }_{0}^{1}\frac{d{S}_{w}}{{P}_{c}^{2}}}=\frac{{\int }_{{S}_{h}}^{{S}_{h}+{S}_{g}-{S}_{gr}}\frac{d{S}_{w}}{{P}_{c}^{2}}}{{\int }_{0}^{1}\frac{d{S}_{w}}{{P}_{c}^{2}}}$$

7 $${k}_{rw}=\frac{{\int }_{0}^{{S}_{h}+{S}_{g}+{S}_{w}}\frac{d{S}_{w}}{{P}_{c}^{2}}-{\int }_{0}^{{S}_{h}}\frac{d{S}_{w}}{{P}_{c}^{2}}-{\int }_{{S}_{h}}^{{S}_{h}+{S}_{g}}\frac{d{S}_{w}}{{P}_{c}^{2}}}{{\int }_{0}^{1}\frac{d{S}_{w}}{{P}_{c}^{2}}}=\frac{{\int }_{{S}_{h}+{S}_{g}}^{{S}_{h}+{S}_{g}+{S}_{w}}\frac{d{S}_{w}}{{P}_{c}^{2}}}{{\int }_{0}^{1}\frac{d{S}_{w}}{{P}_{c}^{2}}}$$

Substituting the general expression for the capillary pressure (Eq. 5) in above two equations gives us:

8 $${k}_{rg}({S}_{w})=\frac{{\int }_{{S}_{h}}^{{S}_{h}+{S}_{g}-{S}_{gr}}\frac{d{S}_{w}}{{P}_{c}^{2}}}{{\int }_{0}^{1}\frac{d{S}_{w}}{{P}_{c}^{2}}}=\frac{\left[c.{\left\{1-b\left(\frac{{S}_{h}+{S}_{g}-{S}_{gr}-{S}_{wr}}{1-{S}_{gr}-{S}_{wr}}\right)\right\}}^{\frac{2}{\lambda }+1}-c.{\left\{1-b\left(\frac{{S}_{h}-{S}_{wr}}{1-{S}_{gr}-{S}_{wr}}\right)\right\}}^{\frac{2}{\lambda }+1}\right]}{\left[c.{\left\{1-b\left(\frac{1-{S}_{wr}}{1-{S}_{gr}-{S}_{wr}}\right)\right\}}^{\frac{2}{\lambda }+1}-c.{\left\{1-b\left(\frac{0-{S}_{wr}}{1-{S}_{gr}-{S}_{wr}}\right)\right\}}^{\frac{2}{\lambda }+1}\right]}$$

where $$c=\frac{1}{\left(\frac{2}{\lambda }+1\right)}.\frac{1}{{P}_{max}^{2}}$$

9 $$\therefore \,{k}_{rg}({S}_{w})=\frac{[{\{(1-{S}_{gr}-{S}_{wr})-b({S}_{h}+{S}_{g}-{S}_{gr}-{S}_{wr})\}}^{\frac{2}{\lambda }+1}-{\{(1-{S}_{gr}-{S}_{wr})-b({S}_{h}-{S}_{wr})\}}^{\frac{2}{\lambda }+1}]}{[{\{(1-b)(1-{S}_{wr})-{S}_{gr}\}}^{\frac{2}{\lambda }+1}-{\{(1-{S}_{gr}-{S}_{wr})+b{S}_{wr}\}}^{\frac{2}{\lambda }+1}]}$$

Similarly,

10 $${k}_{rw}({S}_{w})=\frac{[{\{(1-{S}_{gr}-{S}_{wr})-b({S}_{h}+{S}_{g}+{S}_{w}-{S}_{wr})\}}^{\frac{2}{\lambda }+1}-{\{(1-{S}_{gr}-{S}_{wr})-b({S}_{h}+{S}_{g}-{S}_{wr})\}}^{\frac{2}{\lambda }+1}]}{[{\{(1-{S}_{gr}-{S}_{wr})-b(1-{S}_{wr})\}}^{\frac{2}{\lambda }+1}-{\{(1-{S}_{gr}-{S}_{wr})-b(-{S}_{wr})\}}^{\frac{2}{\lambda }+1}]}$$

$$\because \,{S}_{h}+{S}_{g}+{S}_{w}=1$$, we have

11 $$\therefore \,{k}_{rw}({S}_{w})=\frac{[{\{(1-{S}_{gr}-{S}_{wr})-b(1-{S}_{wr})\}}^{\frac{2}{\lambda }+1}-{\{(1-{S}_{gr}-{S}_{wr})-b({S}_{h}+{S}_{g}-{S}_{wr})\}}^{\frac{2}{\lambda }+1}]}{[{\{(1-b)(1-{S}_{wr})-{S}_{gr}\}}^{\frac{2}{\lambda }+1}-{\{(1-{S}_{gr}-{S}_{wr})+b{S}_{wr}\}}^{\frac{2}{\lambda }+1}]}$$

##### Correction for tortuosity

One of the limitations of the Purcell’s model that assumes a bundle of capillary tubes is that it considers ideal flow in porous media implying ${k}_{rg}+{k}_{rw}=1$. This neglects interfacial phenomena caused by phase trapping and the Jamin effect^55^ in real porous media that usually leads to ${k}_{rg}+{k}_{rw} < 1$^12^. It has been observed that this limitation in the Purcell’s model can be addressed by introducing correction for tortuosity that has a significant impact on the relative permeability^11,12,21,56,57^. Impact of tortuosity is especially amplified at pore-scale because of local variations within the pore network, but it may not be significant at continuum or field-scale where local variations are obscured by the continuum-scale flow. The corrections applied to account for tortuosity is some power of saturation, either two^21,56^ or three^58^. In view of the arbitrary nature of the power that may be specific to each dataset, the tortuosity correction for gas hydrates-bearing sediments is proposed more generally as follows:

12 $${({k}_{ri})}_{correctedfortortuosity}=\mathop{\underbrace{({\beta }_{i}{S}_{i}^{{\eta }_{i}})}}\limits_{correction}.{k}_{ri}$$

where, *β*_*i*_ (>0) and *η*_*i*_ (>0) are empirical parameters $(i=g,w)$ that account for porous media effects such as porosity and tortuosity.

## Results

### Validation

The proposed model is validated using the data reported by Mahabadi *et al*.^23^, who used a 3D pore network model extracted from micro X-ray computed tomography (micro CT) images of gas hydrate-bearing sediments recovered from the Mallik Site^59–61^ to simulate relative permeability of gas and water at three different hydrate saturations. For the purpose of validation, the unknowns are the six fitting parameters $({p}_{e}/{p}_{{\max }},\lambda ,{\beta }_{g},{\beta }_{w},{\eta }_{g},{\eta }_{w})$, while the residual saturations of gas and water, and the hydrate pore morphology are known. The residual saturations are considered known in the validation so as to be consistent with the known values of the experimental data. The residual saturation of water as given in Mahabadi *et al*.^23^ was found to be different for each *S*_*h*_ value.

#### Given information

The actual pore structure extracted from micro CT images of the sediments from the Mallik Site was simplified based on the maximal ball algorithm to a network of spheres connected by tubes, which depicts the pores and pore-throats, respectively, giving the porosity of the sample as 29.3%. Gas hydrate was modeled as pore filling and dissociated by gradually lowering the pressure at the inlet and outlet boundaries from 15 *MPa* to 0.1 *MPa* at a constant temperature of 287 *K*, which causes the decomposition of the gas hydrate (the equilibrium pressure is equal to 13.0 *MPa* at 287 *K*). The process of gas and water flow was simulated for three different *S*_*h*_ (0.2, 0.4, and 0.6) and the relative permeability data is estimated for each *S*_*h*_. The magnitude of residual water saturation (*S*_*wr*_) varies with *S*_*h*_ (*S*_*wr*_ = 0.143, 0.164, 0.211, for *S*_*h*_ = 0.2, 0.4, and 0.6, respectively) because of gas production and water trapping during the preferential flow of gas at locations where the gas pressure is higher than the sum of the water and capillary pressure governed by the Laplace equation for a water-wet surface. The residual gas saturation (*S*_*gr*_) estimated by fitting pore-network simulation results was found to be ~2%. The gas relative permeability data was normalized in Mahabadi *et al*.^23^ and it is used here as it is. To account for spatial heterogeneity in distribution of gas hydrate, five random realizations of each *S*_*h*_ were developed and simulations were performed for all five realizations. Additional details on the pore-scale simulations and relative permeability data used here for validation are given in Mahabadi *et al*.^23^.

#### Curve fitting strategy

Most of the information required to fit relative permeability data using the proposed model are known as discussed above, such as hydrate morphology (pore filling), dissociation temperature, residual saturations (*S*_*wr*_, *S*_*gr*_), except for six parameters $({p}_{e}/{p}_{{\max }},\lambda ,{\beta }_{g},{\beta }_{w},{\eta }_{g},{\eta }_{w})$ that are obtained by fitting the model over relative permeability curves for gas and water at any single *S*_*h*_. The six fitting parameters are estimated by fitting the relative permeability data for only single *S*_*h*_, and the six parameters estimated by fitting relative permeability data for a single *S*_*h*_ can then be used to predict relative permeability at any other *S*_*h*_; this is supported by the mathematical derivation and also by the calculations performed for validation where the same parameters estimated using relative permeability data from a single *S*_*h*_ predict the relative permeability data at other two *S*_*h*_.

**Figure 2:**
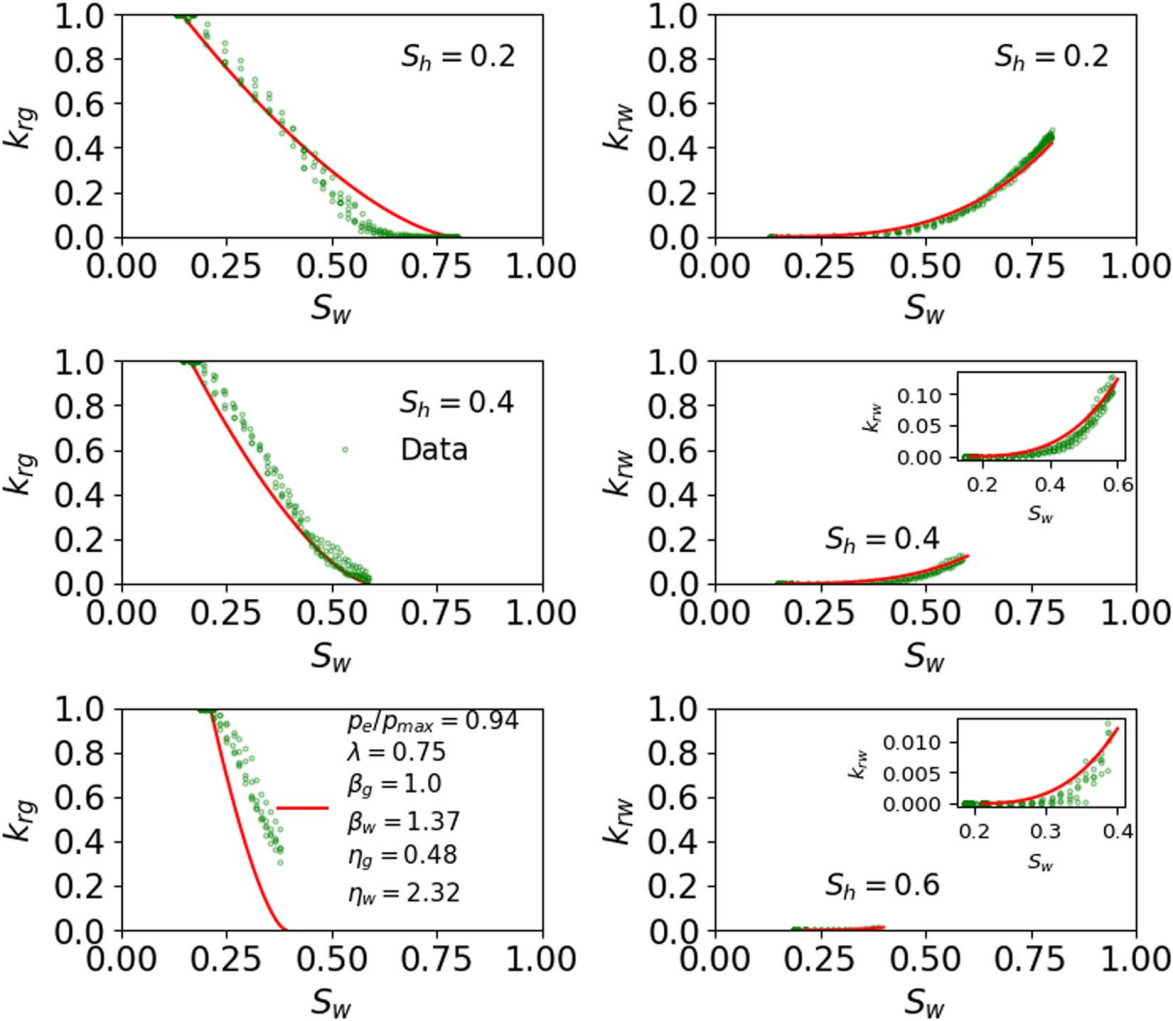
Water and gas relative permeability as estimated using the model for PF gas hydrate and its comparison with pore-scale simulation data (circular markers). The inset in subplot is used to zoom the curve with small values.

Figure 2 shows the relative permeability data reported by Mahabadi *et al*.^23^ in circular markers at three different *S*_*h*_, and the relative permeability predicted by the proposed model in solid red line at each *S*_*h*_. The values of the six model parameters (${p}_{e}/{p}_{{\max }},\,\lambda ,{\beta }_{g},\,{\beta }_{w},\,{\eta }_{g},{\eta }_{w}$) used to match the data are 0.94, 0.75, 1, 1.37, 0.48, and 2.32, respectively. The six unknown parameters of the proposed model are calibrated using experimental data from only single *S*_*h*_ (here ${S}_{h}=0.2$), and the same set of empirical parameters predict relative permeability at the other two *S*_*h*_ (0.4 and 0.6, respectively). The bounds of the unknown parameters and the simple formulation of the equations ensure that the parameter estimation is a well posed problem.

The accuracy of the relative permeability predicted by the model $({k}_{ri}^{model})$ with respect to the relative permeability data $({k}_{ri}^{obs})$ reported by Mahabadi *et al*.^23^ is estimated using a normalized root mean squared error (*RMSE*) for each fluid phase *i* (*i* = *g*, *w*) as follows:

13 $$RMS{E}_{i}=\sqrt{\frac{{\sum }_{k={n}_{1}}^{{n}_{2}}{[{k}_{ri}^{obs}({S}_{w,k})-{k}_{ri}^{model}({S}_{w,k})]}^{2}}{{\sum }_{k={n}_{1}}^{{n}_{2}}1}}$$

14 $$erro{r}_{i}( \% )=\frac{RMS{E}_{i}}{[\max ({k}_{ri}^{obs})-\,\min ({k}_{ri}^{obs})]}\times 100 \% $$

The validation errors for *k*_*rg*_ and *k*_*rw*_ between the model predictions and the data at three different *S*_*h*_ (0.2, 0.4, and 0.6) are 6.8%, 8.7%, 45.5% and 3.8%, 6.7%, 15.9%, respectively. Although the absolute values of the errors may change depending on the relative permeability data available for validation including those at different *S*_*h*_, the general trend of this error sequence would remain the same. This is because the errors at higher *S*_*h*_ would be expected to be higher caused by the amplified effect of tortuosity on gas and water. The effect becomes more pronounced with the increase of pore-scale heterogeneity at higher *S*_*h*_ (discussed below). The novel feature of the proposed model is that it fits the relative permeability curves at each *S*_*h*_ using the same set of parameters that were obtained by fitting the data at any single *S*_*h*_. This also suggests that perturbing a value of any parameter would change the relative permeability curves at all *S*_*h*_, which is shown by the sensitivity analysis of the fitting parameters in the following section.

#### Sensitivity analysis of fitting parameters

To conduct the sensitivity analysis of the fitting parameters two additional curves (dashed-dotted and dotted, both in red) are plotted, representing two different values (low and high) of each parameter, along with the best fitted curve (depicted by solid red line) at each *S*_*h*_. The errors corresponding to each of the three curves (dashed-dotted, dotted, and solid lines), depicting a different value of the fitting parameter, are estimated and presented in Table 1 through Table 2. The fitting parameters that are used in the sensitivity analysis are capillary pressure (*p*_*e*_/*p*_*max*_), pore size distribution index (*λ*), and tortuosity correction $({\beta }_{i},\,{\eta }_{i})$ terms.

**Table 1:** Fitting errors corresponding to each relative permeability curve at all *S*_*h*_ depicting the sensitivity of *p*_*e*_/*p*_*max*_.

*s*_*h*_ (%)	Error (%) in *k*_*rg*_ with RPHCP:			Error (%) in *k*_*rw*_ with RPHCP:		
	Dashed-dotted (*p*_*e*_/*p*_*max*_ = 0.2)	Solid (*p*_*e*_/*p*_*max*_ = 0.94)	Dotted (*p*_*e*_/*p*_*max*_ = 0.99)	Dashed-dotted (*p*_*e*_/*p*_*max*_ = 0.2)	Solid (*p*_*e*_/*p*_*max*_ = 0.94)	Dotted (*p*_*e*_/*p*_*max*_ = 0.94)
20	15.5	6.8	6.9	9.5	3.8	4.2
40	17.2	8.7	8.4	19.2	6.7	7.0
60	50.4	45.5	45.2	37.3	15.9	15.5

**Table 2:** Fitting errors corresponding to each relative permeability curve at all *S*_*h*_ depicting the sensitivity of *η*_*i*_.

*S*_*h*_ (%)	Error (%) in *k*_*rg*_ with RPHCP:			Error (%) in *k*_*rw*_ with RPHCP:		
	Dashed-dotted (*η*_*g*_ = 0.04)	Solid (*η*_*g*_ = 0.48)	Dotted (*η*_*g*_ = 0.96)	Dashed-dotted (*η*_*w*_ = 1.86)	Solid (*η*_*w*_ = 2.32)	Dotted (*η*_*w*_ = 4.29)
20	13.5	6.8	9.2	8.8	3.8	24.8
40	7.2	8.7	15.3	19.1	6.7	30.9
60	29.7	45.5	55.3	48.6	15.9	29.0

##### Capillary pressure term (*P*_*e*_/*P*_max_)

**Figure 3:**
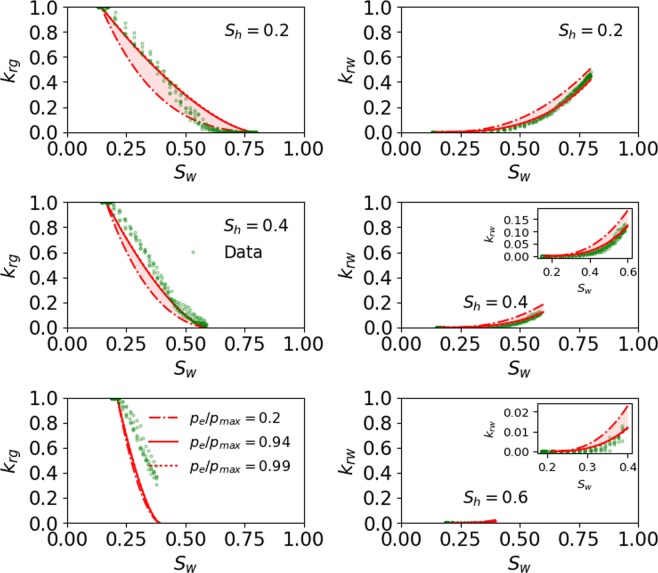
Sensitivity of *p*_*e*_/*p*_*max*_ on prediction of the pore-scale simulation data at all *S*_*h*_. The dashed-dotted and dotted lines represent the lower and higher values of *p*_*e*_/*p*_*max*_. The curve with dotted line almost mimics the best fitted curve (solid red line). The inset in subplot is used to zoom the curve with small values.

Figure 3 shows the sensitivity of *p*_*e*_/*p*_*max*_ on prediction of the pore-scale simulation data at all *S*_*h*_. The errors corresponding to each curve at all *S*_*h*_ are presented in Table 1. Figure 3 shows that porous medium with smaller capillary entry pressure (*p*_*e*_/*p*_*max*_ = 0.2) leads to a smaller *k*_*rg*_ and a larger *k*_*rw*_ when compared to the best fit (*p*_*e*_/*p*_*max*_ = 0.94), whereas the use of a larger capillary entry pressure (*p*_*e*_/*p*_*max*_ = 0.99) almost mimics the best fit because of a minor difference in the capillary entry pressure between the two cases.

##### Pore size distribution index term (*λ*)

**Figure 4:**
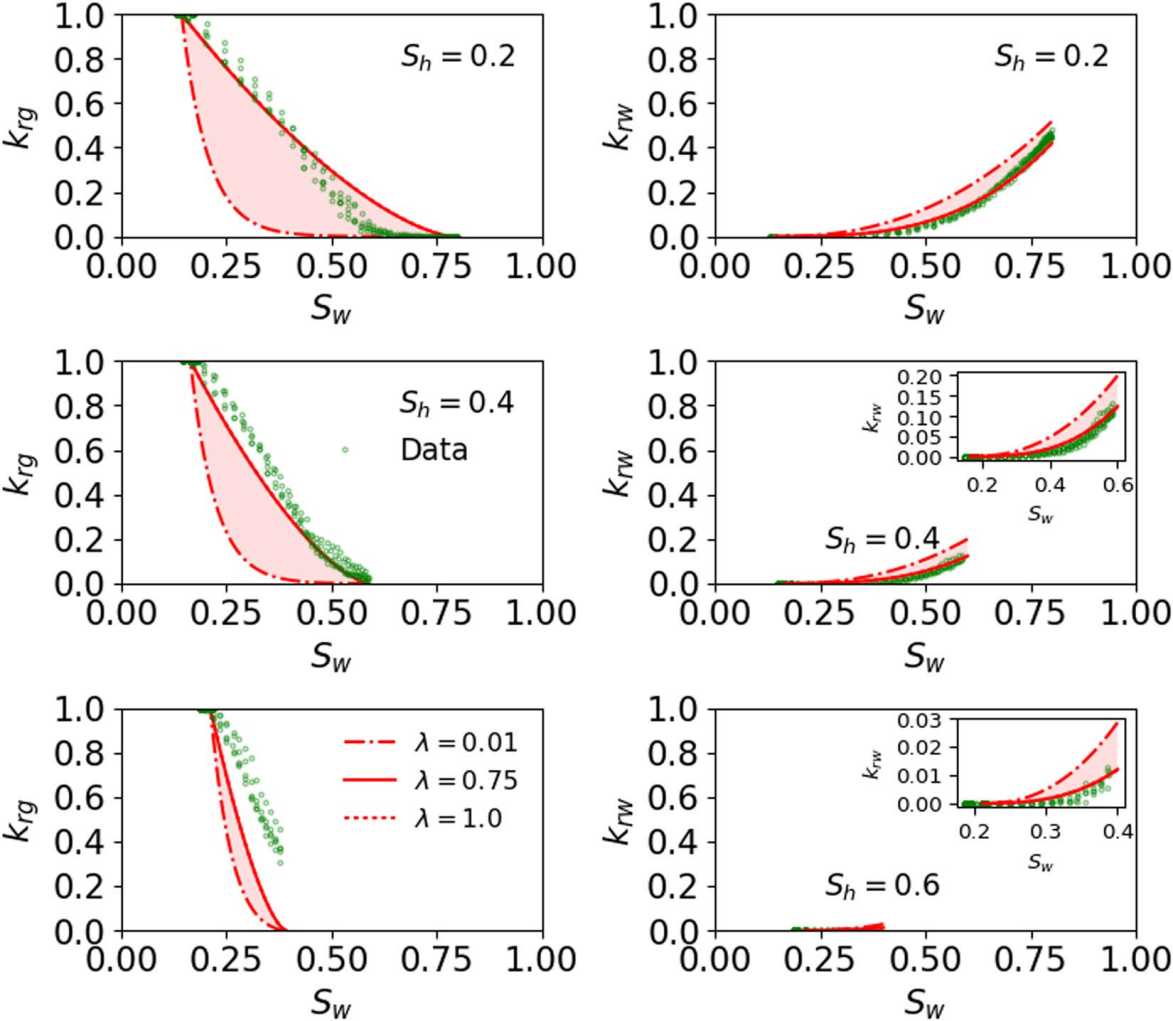
Sensitivity of *λ* on prediction of the pore-scale simulation data at all *S*_*h*_. The dashed-dotted and dotted lines represent the lower and higher values of *λ*. The curve with dotted line almost mimics the best fit (solid red line). The inset in subplot is used to zoom the curve with small values.

In this study, the heterogeneity of porous media is represented in terms of *λ*, so that decrease/increase in heterogeneity is translated into increase/decrease of *λ*^30,35^. Figure 4 shows the sensitivity of *λ* on prediction of the pore-scale simulation data at all *S*_*h*_. The errors corresponding to each curve at all *S*_*h*_ are presented in Table 3. Figure 4 shows that porous medium with larger heterogeneity (*λ* = 0.01) leads to a lower *k*_*rg*_ and a larger *k*_*rw*_ when compared to the best fit (*λ* = 0.75), whereas with the use of smaller heterogeneity (*λ* = 1) almost mimics the best fit indicating that heterogeneity for the best fit case is also small. The heterogeneity of the best fit case is likely small given that the pore scale model by Mahabadi *et al*.^23^ was developed using the simple maximal ball algorithm to a network of spheres connected by tubes.

**Table 3:** Fitting errors corresponding to each relative permeability curve at all *S*_*h*_ depicting the sensitivity of *λ*.

*S*_*h*_ (%)	Error (%) in *k*_*rg*_. with RPHCP:			Error (%) in *k*_*rw*_ with RPHCP:		
	Dashed-dotted (*λ* = 0.01)	Solid (*λ* = 0.75)	Dotted (*λ* = 1)	Dashed-dotted (*λ* = 0.01)	Solid (*λ* = 0.75)	Dotted (*λ* = 1)
20	39.1	6.8	6.8	11	3.8	3.9
40	40.1	8.7	8.6	26.2	6.7	6.8
60	69.5	45.5	45.4	54.0	15.9	15.8

The impact of capillary entry pressure and heterogeneity can be assessed from Figs. 3 and 4, respectively, which show that a porous medium with a larger capillary entry pressure (*p*_*e*_/*p*_*max*_ ≥ 0.94) and smaller heterogeneity $(\lambda \ge 0.75)$ leads to higher *k*_*rg*_ and a smaller *k*_*rw*_. Additionally, the impact of a capillary entry pressure and pore size heterogeneity is significantly more prominent on *k*_*rg*_ than it is on *k*_*rw*_ for ${S}_{h}\le 40 \%$, whereas the impact of these two variables lessens at ${S}_{h}=60 \% .$ The impact of capillary entry pressure on *k*_*rg*_ is larger compared to the impact of pore size heterogeneity on *k*_*rg*_.

##### Tortuosity correction terms $({\beta }_{i},{\eta }_{i})$

**Figure 5:**
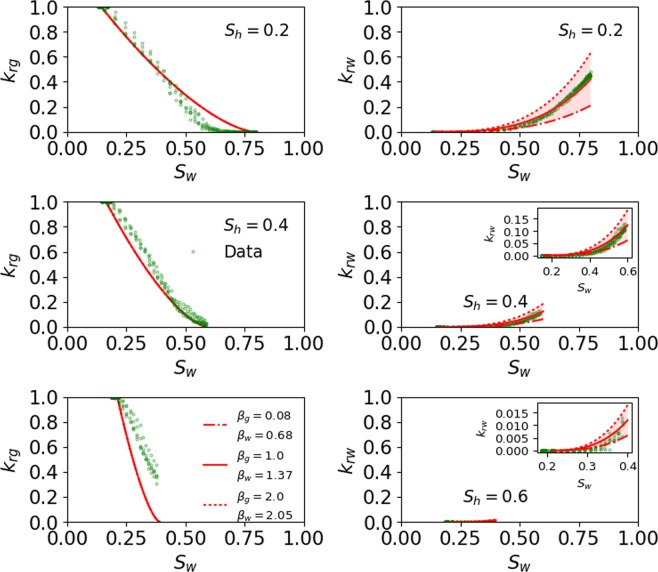
Sensitivity of *β*_*i*_ on prediction of the pore-scale simulation data at all *S*_*h*_. The dashed-dotted and dotted lines represent the lower and higher values of *β*_*i*_. The inset in subplot is used to zoom the curve with small values.

**Figure 6:**
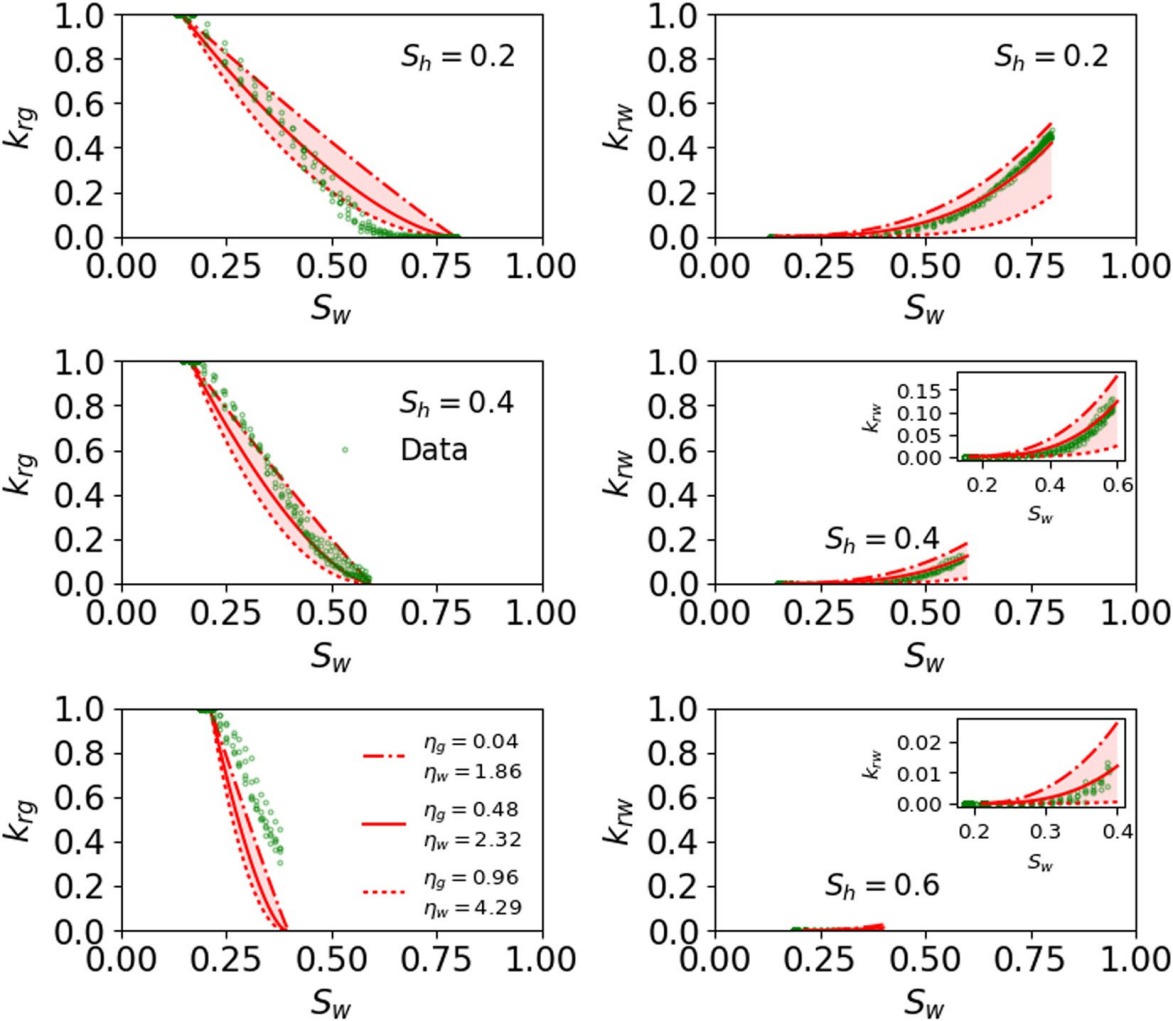
Sensitivity of *η*_*i*_ on prediction of the pore-scale simulation data at all *S*_*h*_. The dashed-dotted and dotted lines represent the lower and higher values of *η*_*i*_. The inset in subplot is used to zoom the curve with small values.

Figures 5 and 6 show the sensitivity of *β*_*i*_ and *η*_*i*_, respectively, on prediction of the pore-scale simulation data at all *S*_*h*_. The errors corresponding to each curve at all *S*_*h*_ are presented in Tables 2 and 4, respectively. The effect of *β*_*g*_ on *k*_*rg*_ is not apparent because the gas relative permeability data was normalized like in Mahabadi *et al*. (2016), which cancels the effect of the *β*_*g*_ term. However, *k*_*rw*_ is not normalized, and therefore, it shows the sensitivity of varying *β*_*w*_.

**Table 4:** Fitting errors corresponding to each relative permeability curve at all *S*_*h*_ depicting the sensitivity of *β*_*i*_.

*S*_*h*_ (%)	Error (%) in *k*_*rg*_ with RPHCP:			Error (%) in *k*_*rw*_ with RPHCP:		
	Dashed-dotted (*β*_*g*_ = 0.08)	Solid (*β*_*g*_ = 1)	Dotted (*β*_*g*_ = 2)	Dashed-dotted (*β*_*w*_ = 0.68)	Solid (*β*_*w*_ = 1.37)	Dotted (*β*_*w*_ = 2.05)
20	6.8	6.8	6.8	19.8	3.8	14.1
40	8.7	8.7	8.7	20.0	6.7	15.5
60	45.5	45.5	45.5	18.6	15.9	24.7

##### Unique parameters set

**Figure 7:**
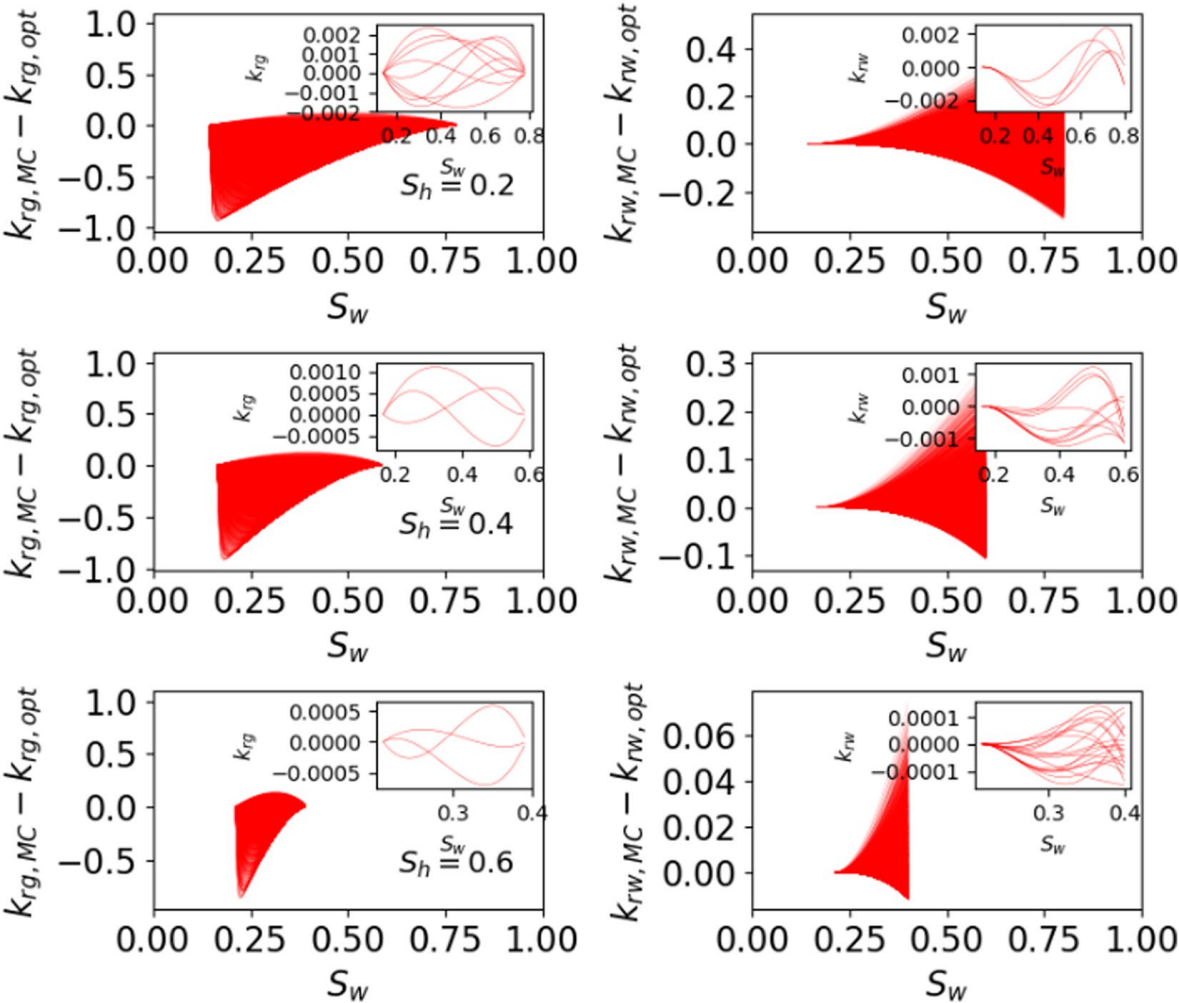
Difference between the relative permeabilities predicted by Monte Carlo simulations $({k}_{ri,MC})$ and the best (optimum) match obtained in validation $({k}_{ri,opt})$ as a function of *S*_*w*_ at three different *S*_*h*_. The inset in subplot is used to zoom near zero to prove that proposed model’s best match exists with only unique parameter values.

To demonstrate unique model output with different set of parameters, Monte-Carlo simulations are performed with 10,000 random sets of six fitting parameters $({p}_{e}/{p}_{{\max }},\lambda ,{\beta }_{g},{\beta }_{w},{\eta }_{g},{\eta }_{w})$, where the range of each parameter is fixed based on its variability as discussed above. Figure 7 shows the difference between the relative permeabilities predicted by Monte Carlo simulations (*k*_*ri*,*MC*_) and the best (optimum) match obtained in section 3.1 $({k}_{ri,opt})$ as a function of *S*_*w*_ at three different *S*_*h*_. Figure 7 shows that there are no two sets of parameters that can give the same output (${k}_{ri,MC}-{k}_{ri,opt}=0$ for same output) for the whole range of *S*_*w*_, or in other words only one set of parameters gives the best match. The non-zero value of ${k}_{ri,MC}-{k}_{ri,opt}$ as a function of *S*_*w*_ (at different *S*_*h*_) in Fig. 7 verifies that the proposed model’s best match exists with only unique parameter values and no two different set of parameters can provide the same match.

### Numerical simulations with proposed model

**Figure 8:**
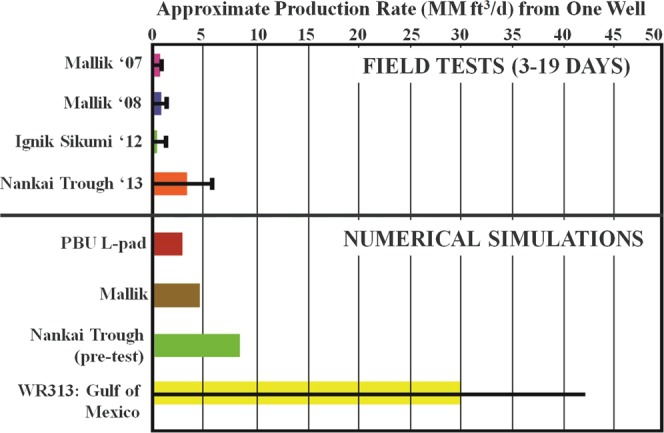
Average (colored bars) and maximum (black line) production rates (after Boswell *et al*.^63^) from gas hydrate accumulations observed at field tests (top half of the figure; duration 3-19 days) and predicted by numerical simulations (bottom half of the figure).

Reliable prediction of a gas hydrate deposit productivity is challenging due to uncertainties in key reservoir parameters and lack of long-term field production tests that would be extremely useful for history-matching and validation of reservoir models. As an example, gas rates predicted from Nankai Trough gas hydrate reservoirs was about two and a half times larger the actual field production rates as shown in Fig. 8. Figure 8 also shows that the rates estimated by numerical simulations for early periods, mimicking the duration of field test, are significantly high, which rationalizes the need for long-term field production tests to assess whether the production from gas hydrate reservoirs can reach commercially viable rates. Although uncertainty and heterogeneity in key petrophysical parameters^62^ remain one of the biggest challenges to conduct accurate simulations of gas production from gas hydrate reservoirs, another important aspect is the use of constitutive models that reliably represent the physics of gas hydrate reservoirs. More specifically, predicting accurate production rates from gas hydrate reservoirs requires elaborated models for relative permeability controlling mass-transport in a producing gas hydrate reservoir. Such models should depend on gas hydrate saturation evolution.

The multiphase relative permeability model proposed in this study is used in numerical simulations to predict gas and water production rates from two gas hydrate reservoirs on the Alaska North Slope (ANS). The production rates predicted using the proposed model are compared with the corresponding output using the B-C relative permeability function. The B-C model is a legacy model that was proposed for use in conventional oil and gas reservoirs, but it has also found its usage in gas hydrate reservoirs because of lack of relative permeability models developed for gas hydrate reservoirs. In that respect, the fixed values of exponents in the B-C model are assumed to account for the average effect of variation in *S*_*h*_ on relative permeability^23^.

#### Gas hydrate reservoirs

##### Kuparuk 7-11-12 reservoir

This gas hydrate reservoir site is located in the western part of the Prudhoe Bay Unit (PBU) of ANS at the Kuparuk 7-11-12 Pad, where a 7-11-12 well was drilled in 1981^64,65^. The well-log data acquired from this well confirmed the occurrence of gas hydrate with large saturations within two zones that are identified as Unit B and Unit D. Further details about the site and the “7-11-12” well can be found in other literature^64,65^.

The 2-D reservoir model of the Unit B was created using the homogeneous representation of the key reservoir properties. The reservoir model uses the homogeneous representation of the key properties and provides three cases with initial *S*_*h*_ equal to 20%, 40%, and 60%. Relevant initial conditions and properties of this reservoir are given in Table 5 and Table 6.

**Table 5:** Reservoir initial condition and properties for the Kuparuk Site.

Parameter	Value
Porosity (fraction)	0.4
*S*_*h*_ (fraction)	0.6/0.4/0.2
Initial Reservoir Pressure (MPa)	8.87 (average)
Initial Reservoir Temperature (°C)	10.52 (average)
Intrinsic permeability (mD)	1000
Brine salinity (ppt)	5

**Table 6:** Reservoir initial condition and properties for the Mt. Elbert Site.

Parameter	Value (averaged)
Porosity (fraction)	0.377
*S*_*h*_ (fraction)	0.586
Initial Reservoir Pressure (MPa)	6.87
Initial Reservoir Temperature (°C)	2.79
Intrinsic permeability (mD)	1100
Brine salinity (ppt)	5

##### Mount Elbert reservoir

**Figure 9:**
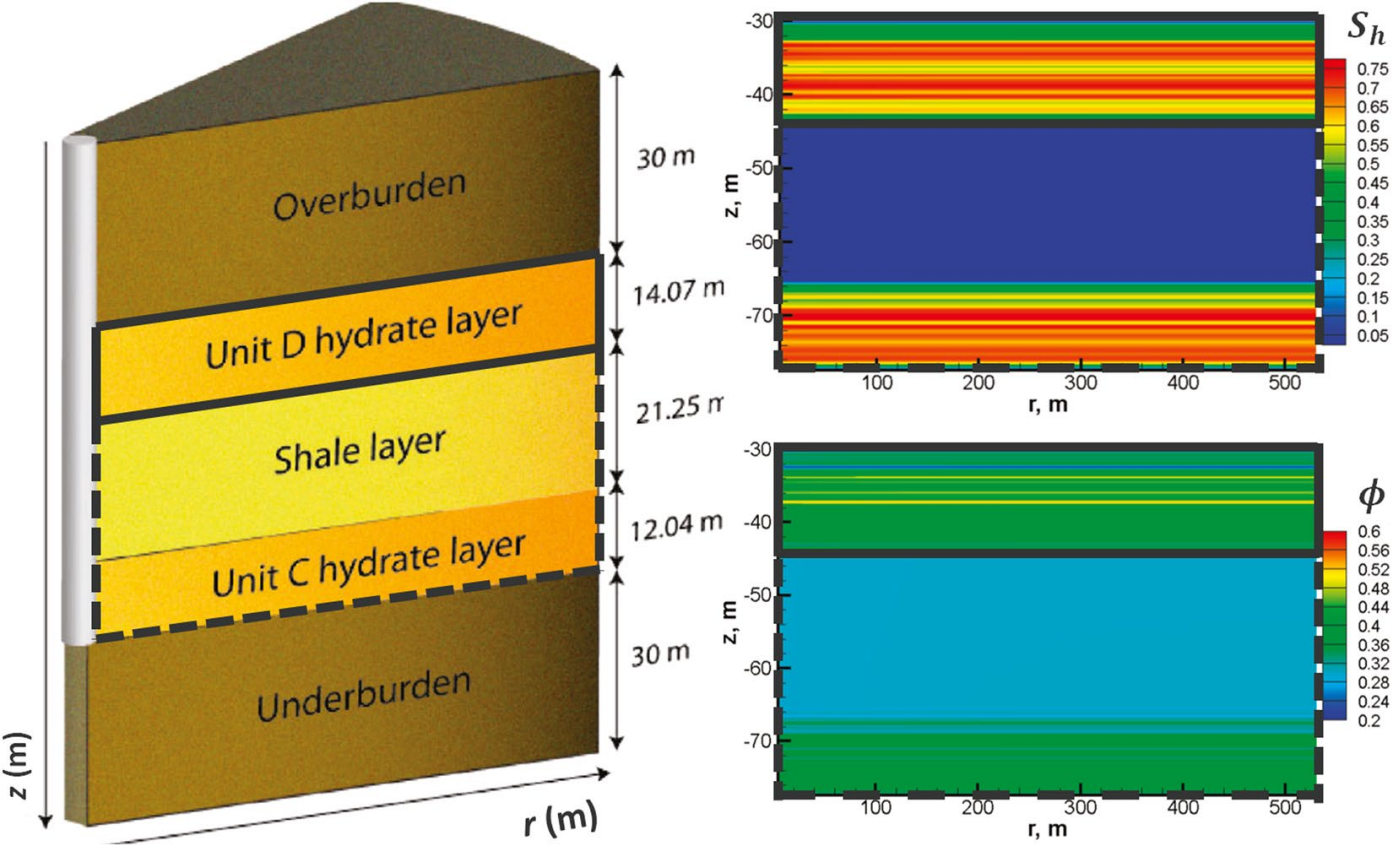
Stratigraphic model of the Mt. Elbert Site (left) with its hydrate saturation (*S*_*h*_) and porosity (*ϕ*) shown on the right (after Myshakin *et al*.^13^). The “cold” Unit D reservoir (bounded by solid black lines) is used for investigation in this study. Relevant initial condition and properties of this reservoir are given in Table 5.

This gas hydrate reservoir site is located in the Milne Point Unit on ANS, where a stratigraphic test well (“Mt. Elbert” stratigraphic test well) was drilled in 2007^66^. A major goal of this well was to assess the response of different downhole well-logging tools in the presence of gas hydrates. As part of this goal, extensive data was collected and interpreted^66^, which confirmed the presence of two gas hydrate-bearing sand zones (Unit D and Unit C). The reservoir model utilizes the heterogeneous representation of the porosity, permeability, and gas hydrate saturation distributions resulting in an initial *S*_*h*_ (average) value of 58.6%. Further details about the site and the “Mt. Elbert” stratigraphic test well can be found elsewhere^13,66^. Numerical simulations are performed for Unit D depicted in Fig. 9 as a part of the schematic stratigraphic model of the Mt. Elbert Site.

#### Numerical simulation results

Numerical simulations of the two reservoirs described above are conducted using the B-C model and the proposed relative permeability model. The gas and water rates/volumes are collected to compare production using the two relative permeability models. For the purpose of consistent comparison the values of exponents in the B-C model are taken within the ranges recommended by Mahabadi *et al*.^23^ for uniformly distributed gas hydrate. The parameters of the B-C and the RPHCP models used in the simulations are listed in Table 7. The fixed empirical parameters for the B-C model assume to take into account the average effect of *S*_*h*_ variation on relative permeability.

**Table 7:** Relative permeability models used in the numerical simulations.

*k*_*r*_ Model	*k*_*w*_	*k*_*g*_	*S*_*wr*_
RPHCP (this study)	Eq. 11	Eq. 9	Variable data from Mahabadi *et al*.^23^
B-C	${\left(\frac{{S}_{w}-{S}_{wr}}{1-{S}_{wr}}\right)}^{{n}_{w}}$; *n*_*w*_ = 3.10	${\left(\frac{{S}_{g}-{S}_{gr}}{1-{S}_{gr}-{S}_{wr}}\right)}^{{n}_{g}}$; *n*_*g*_ = 3.16	0.10

The proposed relative permeability model was implemented in the TOUGH + Hydrate simulator^67^. The numerical simulations were performed using two models with the evolving porous media approach that considers the effective pore space occupied by mobile phases only, or, in other words, includes gas hydrate as a part of the solid matrix. The intrinsic permeability adjustment due to the presence of gas hydrate was estimated using Eq. (3) in Myshakin *et al*.^13^ with *n* = 5.67.

##### Kuparuk Site with 7-11-12 well

**Figure 10:**
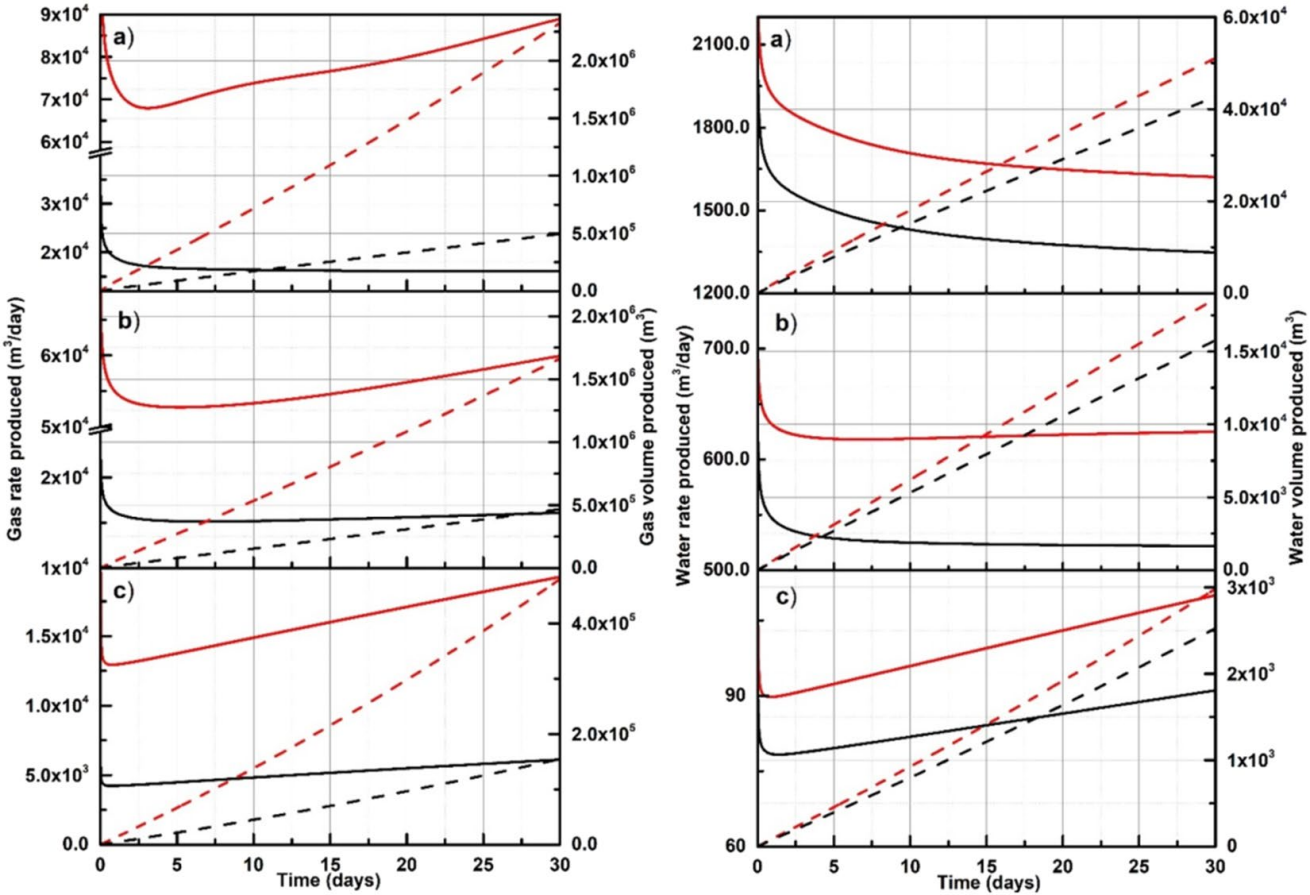
Rates (solid lines) and volumes (dashed lines) for gas (left plots) and water (right plots) produced from the homogeneous reservoir model for the Kuparuk Site using the proposed relative permeability model (red lines) and the B-C model (black lines). The initial *S*_*h*_ in these plots are a) 20%, (**b**) 40%, and (**c**) 60%.

Figure 10 shows the production rates (solid lines) and volumes (dashed lines) for gas (left plots) and water (right plots) predicted from the homogeneous reservoir model for the Kuparuk 7-11-12 Site at three initial gas hydrate saturations (*S*_*h*_ = 20%, 40%, and 60%).

##### Mt. Elbert (Unit D) Site with stratigraphic test well

**Figure 11:**
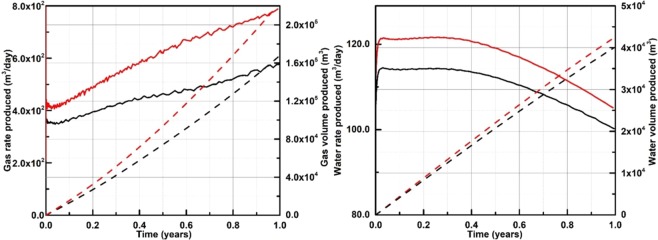
Rates (solid lines) and volumes (dashed lines) for gas (left plot) and water (right plot) produced from the 2-D heterogeneous reservoir model (Unit D) of the Mt. Elbert Site using the proposed relative permeability model (red lines) and the B-C model (black lines).

Figure 11 shows the production performance of the 2-D heterogeneous reservoir model for the Mt. Elbert Site at an initial average gas hydrate saturation of 58.6%. The red lines depict the results computed using the proposed relative permeability model, while the black lines are calculated using the B-C model.

The results presented in Figs. 10 and 11 show large differences in rates and volumes computed using the proposed and the B-C models. That indicates that the use of fixed empirical parameters for the B-C model is a rough approximation when it is applied over a range of *S*_*h*_ in a producing gas hydrate reservoir. The proposed model utilizing the explicit dependency on a *S*_*h*_ value provides the refined prediction of reservoir performance.

##### Computational execution time

Table 8 shows that the CPU run times required to run simulations with the proposed model are very close (a difference of ~0.07 second per iteration, on average, between the two models) to the times taken to run simulations with the B-C model. Therefore, the implementation of the more elaborated expressions for the relative permeability developed in this work (Eqs. 9 and 11) into the simulator leads to small changes in computational times compared to the B-C model.

**Table 8:** Computational execution time per iteration (in seconds) for each case study. The average difference in execution time between the proposed model and the B-C model is ~0.07 seconds per iteration.

*k*_*r*_ Model	Kuparuk			Mount Elbert
	*S*_*h*_ = 20%	*S*_*h*_ = 40%	*S*_*h*_ = 60%	*S*_*h*_ = 58.6% (avg)
RPHCP (this study)	0.574	0.672	0.624	0.459
B-C	0.533	0.616	0.587	0.421

## Discussions

### Quality of the relative permeability validation

Permeability reduction curves in gas hydrate-bearing media exhibit large experimental errors as depicted by the reported data^6–10^. In comparison the prediction errors of the proposed model, ranging from 6.8% to 45.5% for *k*_*rg*_ and from 3.8% to 15.9% for *k*_*rw*_ (Fig. 2), are modest and within an acceptable range. It should be noted that this is the only study so far that provides a match with multiphase relative permeability data at three different *S*_*h*_ and as per our knowledge there is no literature showing fit of existing models that can be used to ascertain the quality of their fit as a reference. The data used in this study for validation were reported by Mahabadi *et al*.^23^ They used them to calibrate empirical parameters of the existing models, but their study did not provide any quantitative or qualitative measure of the quality of their fits.

Unlike the results for *k*_*rw*_, which show almost an ideal match, a large deviation in *k*_*rg*_ has been observed in other reservoirs with hydrocarbons^11,12,58^. A possible reason hypothesized by the noted studies attributes this deviation to tortuosity of gas flow. The deviation in *k*_*rg*_ increases with increase of *S*_*h*_, which could indicate change in gas hydrate morphology^3,41^. A possible reason for the increase in deviation of *k*_*rg*_ at high *S*_*h*_ is possibly due to a shift from pore filling (at intermediate *S*_*h*_ < 40%) to other morphologies (e.g. grain coating, load bearing etc.) at high *S*_*h*_ (>40%)^3^. If the shape of the transformed hydrate morphology at high *S*_*h*_ (~60%) is known, for example, to be grain coating, the fit for *k*_*rg*_ could be improved by applying the model specific to grain coating hydrates at that *S*_*h*_ (derivation available in supplemental material) or even use a combination of two precipitation habits with weighted coefficients.

### Unknown empirical parameters

The unknown parameters in the proposed model (three each for water and gas) have associated physical meaning that accounts for three properties of the rock, namely, capillary pressure (entry and maximum), pore size heterogeneity, and tortuosity experienced by each fluid at gas hydrate-free condition. Although the values of these rock properties may vary with *S*_*h*_, especially with large variation in *S*_*h*_, the mathematical formulation of the proposed model assumes the values of these parameters specifically at gas hydrate-free condition $({S}_{h} \sim 0)$, meaning that their values can also be easily measured experimentally within a gas hydrate-free specimen. In most cases, a capillary pressure function considered as input in reservoir simulations can be used to estimate (*p*_*e*_/*p*_*max*_) assuming that an input capillary pressure table contains the minimum (entry) and maximum possible values for a given reservoir.

### Implications for numerical simulation of gas hydrate reservoirs

The results obtained in Figs. 10 and 11 illustrate that the proposed model can be used to simulate multiphase flow in gas hydrate-bearing medium with dissociating gas hydrate, while not carrying limitations associated with the use of existing models like B-C^37^ or van-Genuchten^22^ as discussed earlier. Significant deviation in the production rates of gas and water for the two reservoirs (presented in Figs. 10 and 11) suggest the important implication of relative permeability, which incorporates the evolving nature of the gas hydrate porous media, as one of the key modeling components in achieving an accurate prediction of the field production rates. The current practice in numerical simulations of gas hydrate reservoirs considers relative permeability that remains unchanged as gas hydrate dissociates, primarily because existing models used in numerical simulations of multiphase flow do not account for variable *S*_*h*_ and, as a result, lack the natural ability to evolve with dissociating gas hydrate medium. Although the B-C model can be customized to discretely account for *S*_*h*_, it would require calibrating empirical parameters at various *S*_*h*_ using experimental data from each of the several *S*_*h*_. This task is a time and resource intensive process as it requires separate experiments at each *S*_*h*_. Moreover, each experiment would require to come up with an empirical equation would most likely depend on the heterogeneity and complexity of the porous medium. Consequently, the dependency of the empirical parameters on *S*_*h*_ deducted using, for example, 20%, 40%, and 60% *S*_*h*_ might not hold true at higher *S*_*h*_ (e.g. 80%), which is found in high quality (large porosity, large intrinsic permeability, and large *S*_*h*_) gas hydrate reservoirs such as Unit B of the Kuparuk Site. Notably, for *S*_*h*_ 80% the residual water saturation would be 23.6%, according to the equation recommended in Mahabadi *et al*.^23^, which means no flow since only 20% of water saturation remains in pore space.

The gas production rates predicted by the proposed model are higher than the production rates predicted by the B-C model. In fact, the gas production rates predicted by the proposed model deviate further apart from the rates predicted by the B-C model as the simulation time progresses, depicting that the impact of relative permeability model, which includes the physics of evolving gas hydrate saturation, can be significantly different than the B-C model on the gas production. It is important to note here that the objective of this work is not to implement a relative permeability model in TOUGH+Hydrate to simulate production rates, but to report one of the first attempts to develop a new relative permeability model for gas hydrate-bearing sediments in general that can be used in lab and field applications.

One might argue that the “original porous media” (OPM) approach^67^ can implicitly account for changes in *S*_*h*_ into the relative permeability model by considering a constant porosity for the medium, such that the changing *S*_*h*_ adjusts the fluid saturations accordingly if the porosity is held constant. Although this approach appears theoretically correct, it is not physically realistic in a way that gas hydrate is a solid phase such that its dissociation changes the void fraction available for the fluid flow, which is defined as porosity. In other words, considering a constant porosity for gas hydrate-bearing media creates a hypothetical concept that is not physically realistic and it bears implications on the fluid flow and solid-mechanics simulations by considering a void fraction (porosity) that is larger than what is physically available for fluid flow in the medium. The “evolving porous media” (EPM) model implemented into TOUGH+Hydrate implies a two-step estimation of effective permeability for a mobile phase. First, it is a reduction of absolute permeability because of the presence of gas hydrate. Second, it is estimation of relative permeability for a mobile phase as if gas hydrate is absent. This means that aqueous phase behaves as if gas hydrate precipitation occurs only in pore space occupied by gas phase; the same statement is true for gas phase. Since gas hydrate cannot simultaneously be present in only aqueous and only in gas-filled pore space, the EPM conceptual limitation is evident. The proposed model is free of such limitations.

## Summary and Conclusions

This study proposed a new relative permeability model for multiphase flow in gas hydrate-bearing porous media. The new model is developed using the Purcell’s approach based on capillary pressure model, which was described by a general empirical equation that is a function of maximum capillary pressure, capillary entry pressure for the wetting phase, pore size distribution index, normalized water saturation, hydrate saturation, residual saturations. Empirical relative permeability models used for multiphase flow in gas hydrate-bearing media were originally developed for conventional oil and gas reservoirs, where the empirical constants depict the porous media with fixed petrophysical properties (e.g. porosity, permeability). However, the effective porosity available for mobile phase flow in gas hydrate-bearing porous media continuously changes during production, therefore, accurate prediction of relative permeability using legacy models would require empirical constants to be a function of *S*_*h*_. That is a time-consuming and resource intensive process as it requires experimental relative permeability data at several *S*_*h*_. The proposed model requires fitting its four empirical parameters $({\beta }_{g},{\beta }_{w},{\eta }_{g},{\eta }_{w})$, besides two non-empirical parameters (*p*_*e*_/*p*_*max*_, *λ*), only once using experimental data from only one *S*_*h*_, and the same set of empirical parameters can predict relative permeability at any other *S*_*h*_. The model also accounts for the effect of pore-size distribution in the rock matrix, which is not possible with the models currently used for numerical simulations of gas hydrate reservoirs.

The numerical simulations performed using the proposed model take similar time as the time taken by the B-C model for the two reservoirs studied. Reservoir productivity predicted by employing the proposed model is different than that computed by the B-C model using fixed empirical parameters for gas and water relative permeability. That manifests an approximate nature of the B-C model applied over a range of *S*_*h*_ values in comparison with the proposed model that uses the explicit dependency on *S*_*h*_ to provide the refined prediction of reservoir performance.

### Disclaimer

This work was funded by the Department of Energy, National Energy Technology Laboratory, an agency of the United States Government, through a support contract with Leidos Research Support Team (LRST). Neither the United States Government nor any agency thereof, nor any of their employees, nor LRTS, nor any of their employees, makes any warranty, expressed or implied, or assumes any legal liability or responsibility for the accuracy, completeness, or usefulness of any information, apparatus, product, or process disclosed, or represents that its use would not infringe privately owned rights. Reference herein to any specific commercial product, process, or service by trade name, trademark, manufacturer, or otherwise, does not necessarily constitute or imply its endorsement, recommendation, or favoring by the United States Government or any agency thereof. The views and opinions of authors expressed herein do not necessarily state or reflect those of the United States Government or any agency thereof.

## Supplementary information

Supplementary Information.

## Data Availability

All data generated or analyzed during this study are included in this published article (and the Appendix).
