# Supplementary material for: A Novel Relative Permeability Model for Gas and Water Flow in Hydrate-Bearing Sediments With Laboratory and Field-Scale Application
Source: Sci Rep. 2020 Mar 30;10:5697. doi: 10.1038/s41598-020-62284-5 (PMC7105472; doi:10.1038/s41598-020-62284-5)
Supplement: Supplementary file 1 — Supplementary Information. [file 41598_2020_62284_MOESM1_ESM.docx]

# A Novel Relative Permeability Model for Gas and Water Flow in Hydrate-Bearing Sediments With Laboratory and Field-Scale Application

Harpreet Singh^1*^, Evgeniy M. Myshakin,^1,2^ Yongkoo Seol^1^**

^1^National Energy Technology Laboratory, Morgantown, WV, USA

^2^LRST, 626 Cochrans Mill Road, Pittsburgh, PA

*harpreet.singh@utexas.edu

**yongkoo.seol@netl.doe.gov

## Appendix

### A.1. Grain Coating (GC) Hydrates

Grain coating hydrates coat the pore walls, which when visualized in cylindrical pores may look as shown in Figure A-1. This type of hydrate morphology leaves an open space in the middle of the pore occupied by water and gas as shown by the sketch in Figure A-1. Conceptual model depicting cross-section of GC hydrates with fluids, and its continuum-scale representation in porous media with matrix (grains) are shown in Figure A-1(a) and Figure A-1(b), respectively.


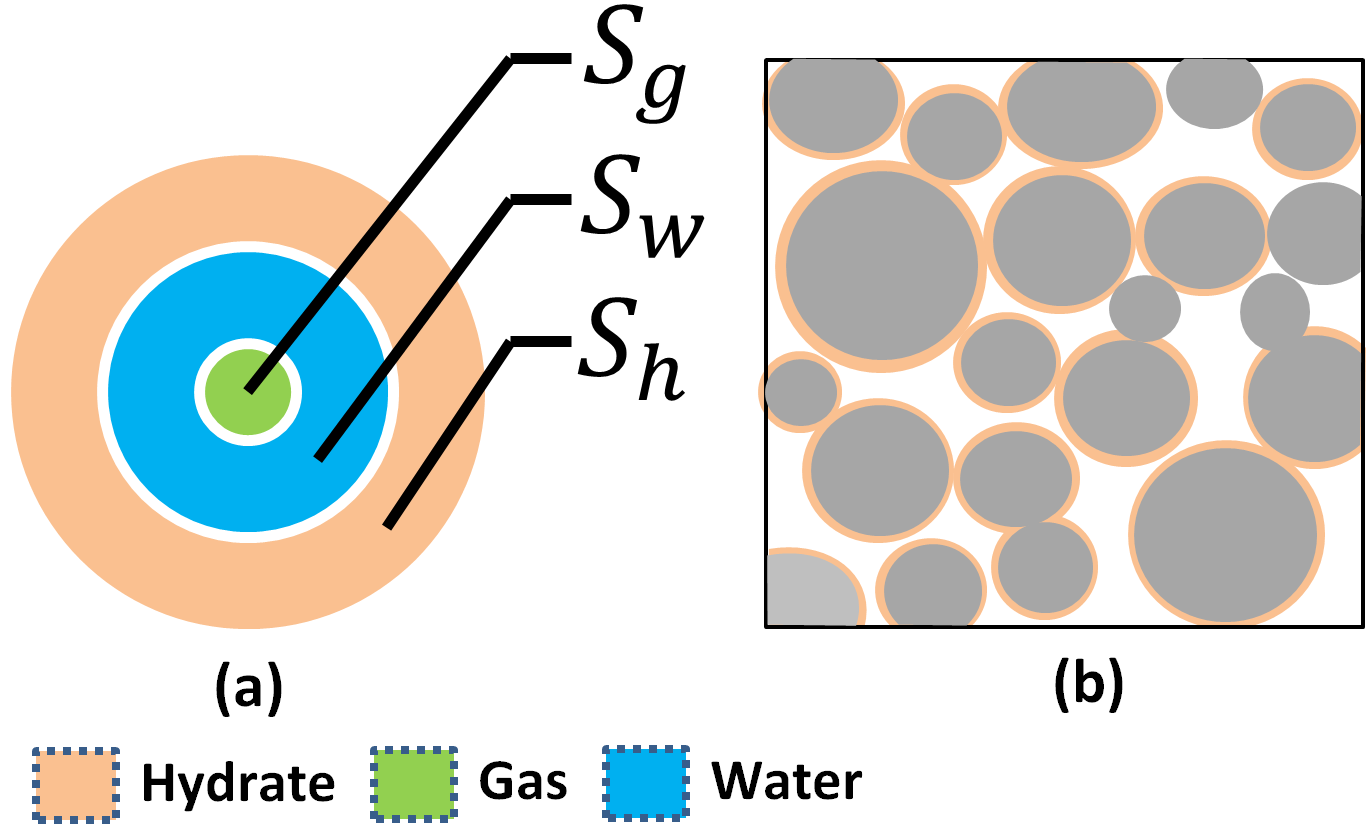


Figure A-1: A sketch depicting (a) a simplified capillary-shaped cross-section for grain coating hydrate with fluids, and (b) its continuum-scale representation in porous media with matrix (grains) in gray color. The boundary conditions shown by this illustration are independent of the pore shape.

Gas and water relative permeability are obtained by using the boundary conditions, independent of the pore shape, from Figure A-1(a) in the limits of the integral as follows.

##### Relative Permeability Model

$k_{rg}=\frac{\int_{0}^{S_{g}-S_{gr}} \frac{dS_{w}}{P_{c}^{2}}}{\int_{0}^{1} \frac{dS_{w}}{P_{c}^{2}}}$ (1)

$k_{rw}=\frac{\int_{0}^{S_{g}+S_{w}} \frac{dS_{w}}{P_{c}^{2}}-\int_{0}^{S_{g}} \frac{dS_{w}}{P_{c}^{2}}}{\int_{0}^{1} \frac{dS_{w}}{P_{c}^{2}}}=\frac{\int_{S_{g}}^{S_{g}+S_{w}} \frac{dS_{w}}{P_{c}^{2}}}{\int_{0}^{1} \frac{dS_{w}}{P_{c}^{2}}}$ (2)

Substituting the general expression for the capillary pressure in above two equations gives us:

$k_{rg}\left( S_{w} \right)=\frac{\left[ \left\{ \left( 1-S_{gr}-S_{wr} \right)-b\left( \underline{S_{g}-S_{gr}}-S_{wr} \right) \right\}^{\frac{2}{\lambda}+1}-\left\{ \left( 1-S_{gr}-S_{wr} \right)-b\left( \underline{0}-S_{wr} \right) \right\}^{\frac{2}{\lambda}+1} \right]}{\left[ \left\{ \left( 1-b \right)\left( 1-S_{wr} \right)-S_{gr} \right\}^{\frac{2}{\lambda}+1}-\left\{ \left( 1-S_{gr}-S_{wr} \right)+bS_{wr} \right\}^{\frac{2}{\lambda}+1} \right]}$ (3)

$\therefore k_{rg}\left( S_{w} \right)=\frac{\left[ \left\{ \left( 1-S_{gr}-S_{wr} \right)-b\left( S_{g}-S_{gr}-S_{wr} \right) \right\}^{\frac{2}{\lambda}+1}-\left\{ \left( 1-S_{gr}-S_{wr} \right)+bS_{wr} \right\}^{\frac{2}{\lambda}+1} \right]}{\left[ \left\{ \left( 1-b \right)\left( 1-S_{wr} \right)-S_{gr} \right\}^{\frac{2}{\lambda}+1}-\left\{ \left( 1-S_{gr}-S_{wr} \right)+bS_{wr} \right\}^{\frac{2}{\lambda}+1} \right]}$ (4)

$\therefore k_{rw}\left( S_{w} \right)=\frac{\left[ \left\{ \left( 1-S_{gr}-S_{wr} \right)-b\left( S_{g}+S_{w}-S_{wr} \right) \right\}^{\frac{2}{\lambda}+1}-\left\{ \left( 1-S_{gr}-S_{wr} \right)-b\left( S_{g}-S_{wr} \right) \right\}^{\frac{2}{\lambda}+1} \right]}{\left[ \left\{ \left( 1-b \right)\left( 1-S_{wr} \right)-S_{gr} \right\}^{\frac{2}{\lambda}+1}-\left\{ \left( 1-S_{gr}-S_{wr} \right)+bS_{wr} \right\}^{\frac{2}{\lambda}+1} \right]}$ (5)

The hydrate morphology shown by Figure A-1 is independent of the pore shape, although they are shown using cylindrical pore shapes for the purpose of illustration.

### A.2. Sensitivity of Hydrate Morphology on Relative Permeability

In this section, the fitting parameters for the best match obtained in the validation section are fixed in order to investigate the impact of hydrate morphology on relative permeability of gas and water. Also, the residual saturations of gas and water are assumed fixed (0.10 and 0.20, respectively) at all $S_{h}$. The values assumed for residual saturations depict a water-wet medium where water has a higher residual saturation than the residual saturation of the non-wetting phase (gas) that exists in larger pores compared to the wetting phase.

Sensitivity of hydrate morphology on relative permeability is assessed using three different morphologies, which are i) pore filling, ii) combination of pore filling and grain coating with 50% each, and iii) grain coating. The relative permeability for a combination of pore filling and grain coating with 50% each is calculated using the relative permeability of two ideal hydrate pore morphologies as follows ^17^:

$k_{ri}=\left[ \left\{ \left( k_{ri} \right)_{PF} \right\}^{x_{PF}}\times\left\{ \left( k_{ri} \right)_{GC} \right\}^{1-x_{PF}} \right]$ (6)

Here, $x_{PF}$ and ($1-x_{PF}$) represent the fraction of pore filling hydrates and the fraction of grain coating hydrates, respectively. Most gas hydrates in the field are composed of morphologies that are more complex than their ideal assumptions of PF and GC, and the case used here with a combination of two ideal hydrate morphologies is an attempt to mimic one such complex morphology. The fluid distribution in this analysis assumes the water as the wetting phase and gas as the non-wetting phase.

The sensitivity of hydrate morphology on $k_{rw}$ and $k_{rg}$ is investigated at three different hydrate saturations ($S_{h}=0.2, 0.4, 0.6$), and it is analyzed systematically for four different scenarios that account for varying capillary entry pressure and rock heterogeneity; these four scenarios are: i) low capillary entry pressure and high heterogeneity, ii) low capillary entry pressure and low heterogeneity, iii) high capillary entry pressure and high heterogeneity, and iv) high capillary entry pressure and low heterogeneity.

#### A.2.1. Low $\boldsymbol{p}_{\boldsymbol{e}}\mathbf{/}\boldsymbol{p}_{\boldsymbol{max}}$ and Low $\boldsymbol{\lambda}$

Results in Figure A-2 show that at low capillary entry pressure and high heterogeneity, the flow of gas is negligible ($k_{rg}\approx0$) over the entire range of $S_{w}$ and for all types of hydrate morphologies. Figure A-2 also shows that the flow of water is significant in PF hydrates, but it is negligible ($k_{rw}\approx0$) over the entire range of $S_{w}$ in GC hydrates and in hydrates with a mixed morphology of PF and GC.


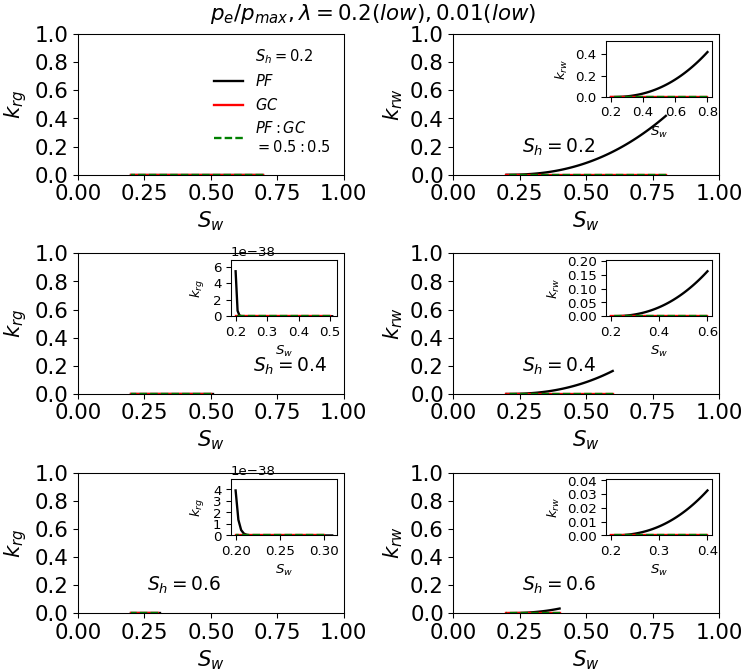


Figure A-2: $k_{rg}$ and $k_{rw}$ (at 20%, 40% and 60% $S_{h}$) for three different hydrate morphologies: i) PF shown by solid black line, ii) GC shown by solid red line, and iii) a combination of PF and GC with 50% each shown by dashed green line. The sensitivity of hydrate morphology on relative permeability is shown here for a scenario with low capillary entry pressure and high heterogeneity.

#### A.2.2. Low $\boldsymbol{p}_{\boldsymbol{e}}\mathbf{/}\boldsymbol{p}_{\boldsymbol{max}}$ and High $\boldsymbol{\lambda}$

Results in Figure A-3 show that at low capillary entry pressure and low heterogeneity, both the flow of gas and water are significantly affected by the type of hydrate morphology at all $S_{h}$. Figure A-3 shows that the relative decrease in $k_{rg}$ and $k_{rw}$with increasing $S_{h}$ is sharpest in GC hydrates followed by hydrates with a mixed morphology, and least in the case of PF hydrates.


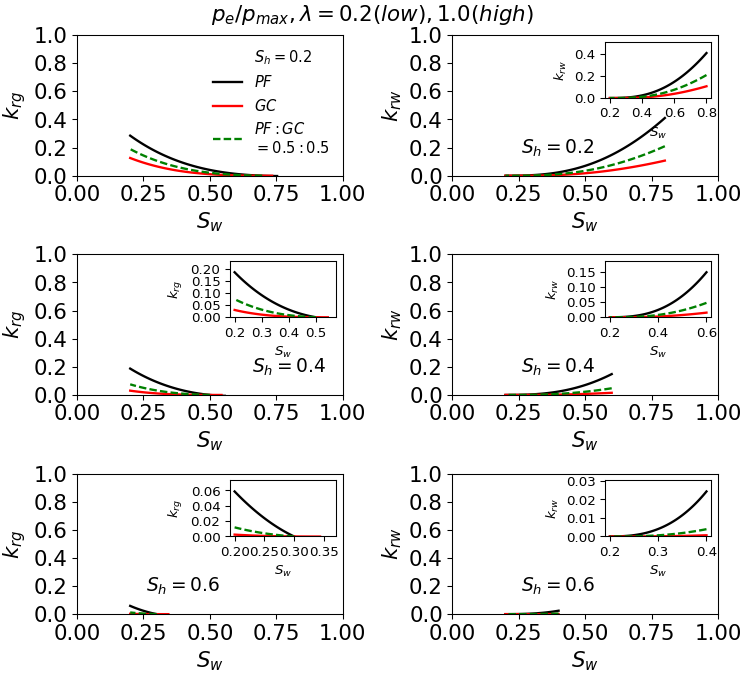


Figure A-3: $k_{rg}$ and $k_{rw}$ (at 20%, 40% and 60% $S_{h}$) for three different hydrate morphologies: i) PF shown by solid black line, ii) GC shown by solid red line, and iii) a combination of PF and GC with 50% each shown by dashed green line. The sensitivity of hydrate morphology on relative permeability is shown here for a scenario with low capillary entry pressure and low heterogeneity.

#### A.2.3. High $\boldsymbol{p}_{\boldsymbol{e}}\mathbf{/}\boldsymbol{p}_{\boldsymbol{max}}$ and Low $\boldsymbol{\lambda}$

Results in Figure A-4 with high capillary entry pressure and high heterogeneity depict similar behavior as the results with low capillary entry pressure and low heterogeneity of Figure A-3.


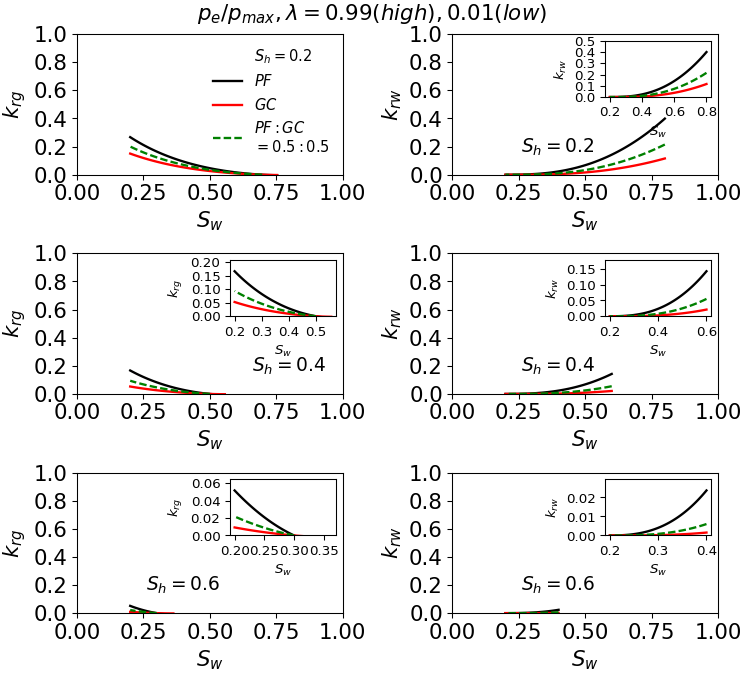


Figure A-4: $k_{rg}$ and $k_{rw}$ (at 20%, 40% and 60% $S_{h}$) for three different hydrate morphologies: i) PF shown by solid black line, ii) GC shown by solid red line, and iii) a combination of PF and GC with 50% each shown by dashed green line. The sensitivity of hydrate morphology on relative permeability is shown here for a scenario with high capillary entry pressure and high heterogeneity.

#### A.2.4. High $\boldsymbol{p}_{\boldsymbol{e}}\mathbf{/}\boldsymbol{p}_{\boldsymbol{max}}$ and High $\boldsymbol{\lambda}$

Results in Figure A-5 show that at high capillary entry pressure and low heterogeneity, the flow of gas is unaffected by the type of hydrate morphology such that for any hydrate saturation, $k_{rg}$ overlaps for all three types of hydrate morphologies. Although Figure A-5 shows that hydrate morphology has minor to moderate impact on $k_{rw}$, the lowest $k_{rw}$ among the three different hydrate morphologies is for GC hydrates, implying that the flow of water is impeded most by the GC hydrates.


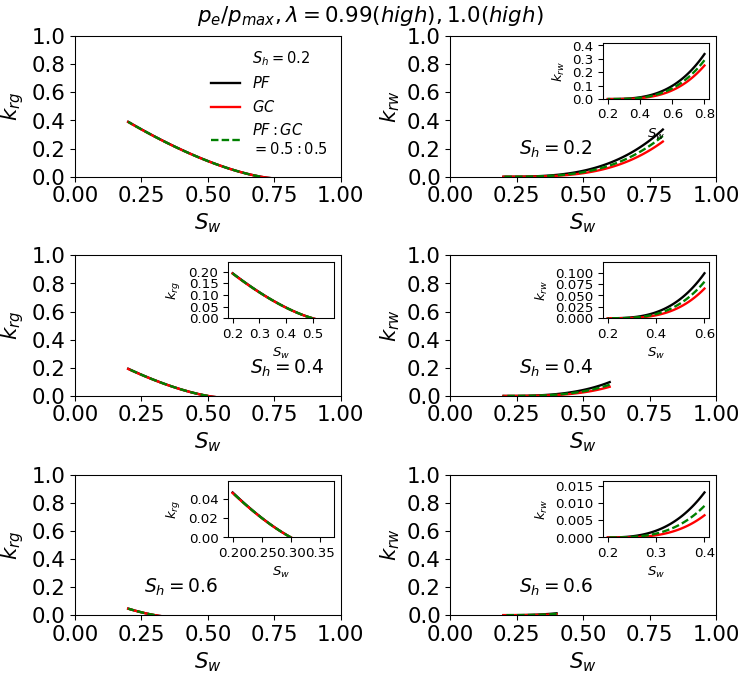


Figure A-5: $k_{rg}$ and $k_{rw}$ (at 20%, 40% and 60% $S_{h}$) for three different hydrate morphologies: i) PF shown by solid black line, ii) GC shown by solid red line, and iii) a combination of PF and GC with 50% each shown by dashed green line. The sensitivity of hydrate morphology on relative permeability is shown here for a scenario with high capillary entry pressure and low heterogeneity.
